# Peptidomic Identification of Serum Peptides Diagnosing Preeclampsia

**DOI:** 10.1371/journal.pone.0065571

**Published:** 2013-06-19

**Authors:** Qiaojun Wen, Linda Y. Liu, Ting Yang, Cantas Alev, Shuaibin Wu, David K. Stevenson, Guojun Sheng, Atul J. Butte, Xuefeng B. Ling

**Affiliations:** 1 Department of Surgery, Stanford University, Stanford, California, United States of America; 2 Department of Pediatrics, Stanford University, Stanford, California, United States of America; 3 Lab for Early Embryogenesis, RIKEN Center for Developmental Biology, Chuo-Ku, Kobe, Hyogo, Japan; Institute of Zoology, Chinese Academy of Sciences, China

## Abstract

We sought to identify serological markers capable of diagnosing preeclampsia (PE). We performed serum peptide analysis (liquid chromatography mass spectrometry) of 62 unique samples from 31 PE patients and 31 healthy pregnant controls, with two-thirds used as a training set and the other third as a testing set. Differential serum peptide profiling identified 52 significant serum peptides, and a 19-peptide panel collectively discriminating PE in training sets (n = 21 PE, n = 21 control; specificity = 85.7% and sensitivity = 100%) and testing sets (n = 10 PE, n = 10 control; specificity = 80% and sensitivity = 100%). The panel peptides were derived from 6 different protein precursors: 13 from fibrinogen alpha (FGA), 1 from alpha-1-antitrypsin (A1AT), 1 from apolipoprotein L1 (APO-L1), 1 from inter-alpha-trypsin inhibitor heavy chain H4 (ITIH4), 2 from kininogen-1 (KNG1), and 1 from thymosin beta-4 (TMSB4). We concluded that serum peptides can accurately discriminate active PE. Measurement of a 19-peptide panel could be performed quickly and in a quantitative mass spectrometric platform available in clinical laboratories. This serum peptide panel quantification could provide clinical utility in predicting PE or differential diagnosis of PE from confounding chronic hypertension.

## Introduction

Preeclampsia (PE) complicates about 5% of all pregnancies worldwide and is a major cause of maternal, fetal and neonatal morbidity and mortality, especially in developing nations [1], [2]. It is a potentially dangerous complication of the second half of pregnancy, labor, or early period after delivery, characterized by hypertension, abnormal amounts of protein in the urine, and other systemic disturbances. PE currently has little effective therapy, though it largely resolves after placenta and fetus delivery [3]. PE is one of the most common reasons for induced preterm delivery [4].

The use of biofluid (e.g. serum or urine) for the analysis of the naturally occurring peptidome (MW<4000) as a source of biomarkers has been reported in different diseases [5]–[9]. For clinical application, mass spectrometry-based profiling of naturally occurring peptides can provide an extensive inventory of serum peptides derived from either high-abundant endogenous circulating proteins or cell and tissue proteins [10]. These peptides are usually soluble, and stable from endogenous proteases or peptidases, and can be directly used for liquid chromatography-mass spectrometry (LC/MS) analysis without additional manipulation (e.g. tryptic digests). However, a serum peptidomics based approach has not been attempted for the discovery of PE biomarkers.

We hypothesized that there would be differential serum peptidomic signatures reflective of a PE-specific alteration of proteolytic and anti-proteolytic pathways. Our peptidomics-based discovery and subsequent validation yielded 19 unique serum peptides differing between PE and control subjects. These peptide biomarkers, collectively as a panel, can effectively assess PE.

## Materials and Methods

### Specimen collection and preprocessing

To identify the PE related peptide sequences, case and control cohorts were constructed to match gestational age, ethnicity, and parity. Serum specimens from 62 pregnant women (PE n = 31, control n = 31) were purchased from ProMedDX Inc. (Norton, MA 02766, http://www.promeddx.com). The PE patients were diagnosed with preeclampsia characterized by both hypertension and proteinuria. As shown in Table 1, all of the 31 PE patients had both hypertension and proteinuria; 41.9% of them had headache; 22.6% of them had edema; and 25.8% of them had other additional symptoms. The 62 samples were divided into two datasets randomly: the training set (n = 21 case group, n = 21 control group); the testing set (n = 10 case group, n = 10 control group). The demographics on the 2 sets (training and testing) were summarized in Table 2, which compares the ethnicity, age and gestation delivery time of the case and control samples (continuous variable: two-tailed Mann-Whitney U test; categorical analysis: Fisher's exact test).

**Table 1:** PE patients' presenting signs and symptoms.

Presenting Signs and Symptoms	Number (percentage)
Hypertension	31 (100%)
Proteinuria	31 (100%)
Headache	13 (41.9%)
Edema	7 (22.6%)
Others	8 (25.8%)

**Table 2:** Demographics.

	Training data			Testing data			Overall
Characteristic	PE	control	*p* value	PE	control	*p* value	*p* value
	n = 21 (50%)	n = 21 (50%)		n = 10 (50%)	n = 10 (50%)		
Ethnicity			**0.512**			**0.164**	**0.286**
African American	6 (28.6%)	5 (23.8%)		1 (10%)	4 (40%)		
Asian	2 (9.5%)	0 (0%)		0 (0%)	0 (0%)		
Hispanic	11 (52.4%)	15 (71.4%)		7 (70%)	6 (60%)		
Other	2 (9.5%)	1 (4.8%)		2 (20%)	0 (0%)		
Age (year)							
median (IQR)	24 (19,32)	24 (20,29)	**0.95**	23 (20,32)	24 (19,26)	**1**	**0.916**
Week of gestation							
median (IQR)	36 (33,37)	33 (28,36)	**0.077**	33.5 (28,37)	37.5 (35,38)	**0.087**	**0.772**

Serum peptides were prepared as previously described in [7]. Serum samples were processed by centrifugal filtration at 3000× g for 20 min at 10°C through Amicon Ultra centrifugal filtration devices (10 kDa cutoff) (Millipore, Bedford, MA) preequilibrated with 10 ml Milli-Q water. The filtrate (serum peptidome) containing the low MW naturally occurring peptides was processed with Waters Oasis HLB Extraction Cartridges (Waters Corporation, Milford, MA), and extracted with ethyl acetate. The serum peptide samples were quantified by the 2,4,6-trinitrobenzenesulfonic acid (TNBS) assay, as described in [11]. Lyophilized human serum peptide samples were reconstituted in 2% acetonitrile with 0.1% formic acid and separated on a Paradigm MS4 liquid chromatography system (Michrom BioResources, Auburn, CA) with a 60 min linear gradient of 5–95% buffer A to B (buffer A: 2% acetonitrile with 0.1% formic acid in H_2_O, buffer B: 90% acetonitrile with 0.1% formic acid in H_2_O) at a flow rate of 2 µl/min using a 0.2×50 mm 3 µ 200 Å Magic C18AQ column (Michrom BioResources, Auburn, CA). Each randomized sample run was followed by a 60 min wash run. The fractionated peptides were directly applied to an LTQ ion trap mass spectrometer (Thermo Fisher Scientific, San Jose, CA) equipped with a Fortis tip mounted nano-electrospray ion source (AMR, Tokyo, Japan). The Fortis tip is with 150 µm outside diameter (OD) and 20 µm inside diameter (ID), which can be used with flow rates between 200–2000 nl/min. The electrospray voltage was set at 1.8 kV. Each full MS scan with a mass range of 400–2000 *m/z* was followed by two data-dependent scans of the two most abundant ions observed in the first full MS scan. MS/MS spectra were generated for the highest peak in each scan with the relative collision energy for MS/MS set to 35%. Raw MS/MS data were preprocessed, as previously described [12], before further statistical analysis. Peptide protein identification was search against the human SwissProt database as previously described. At first, the intensity values of the same peptides in the same proteins were summed up across different fractions for each sample. Therefore, each peptide in one sample has one intensity value, which was later normalized by the total intensity value of all peptides found in the sample.

### Feature selection to identify discriminative PE serum peptide biomarkers

612 peptides, across all samples, were identified by MS and MS/MS steps and chosen as the biomarker candidates. Significance analysis of microarrays (SAM [13]) was used to calculate *d*-scores indicating the relative positive (increased) and negative (decreased) changes in abundance of these serum peptides in PE subjects in comparison to control subjects. SAM calculated a minimal false discovery rate (*q* value) for significance.

A shrunken centroid algorithm called predictive analysis of microarrays (PAM [14]) was used to find and construct a PE-specific serum peptide panel. 42 samples, balanced in PE and control samples, were randomly selected as the training data of PAM, and the rest 20 samples were used as the testing data. With the training data, training and 100 repeated random sub-sampling cross validation was used to train the PAM model, select the significant features for the diagnostic panel and estimate the prediction error. A threshold was used in the PAM algorithm to control the number of shrunken centroids. A larger threshold will result in a smaller number of shrunken centroids. Generally, as the number of shrunken centroids, namely, selected biomarkers, increases, the prediction error of both the training samples and testing samples will decrease. The estimated PE score of each sample was computed based on the predicted probability of the PAM model (19-peptide panel). In PAM algorithm, a sample was predicted as a PE sample if the score was larger than 0.5. The predictive performance of each biomarker panel analysis was evaluated by sensitivity and specificity analysis.

### ELISA assays validating PE marker candidates

ELISA assays were performed using commercial kits following vendors' instructions. All assays were performed to measure serum levels of placental growth factor (PIGF), R&D system Inc. (MN, US) and soluble fms-like tyrosine kinase (sFlt-1), R&D system Inc.

## Results

### Sample Qualification with sFlt-1 and PIGF Analysis

**Figure 1:**
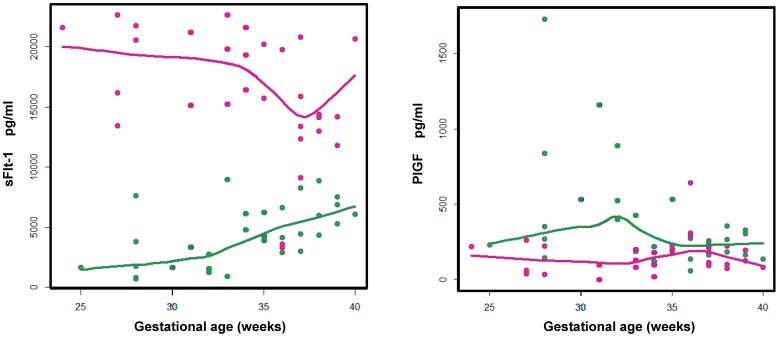
The serum concentrations of sFlt-1 (left) and PIGF (right) as a function of the gestation. For either PE (red) or control (green) data points, a loess curve was fitted to represent the overall trend of biomarker serum abundance as a function of gestation.

Elevated soluble sFlt-1 and decreased PIGF levels are suggested in the pathogenesis of PE [15]–[21], and the sFlt-1/PIGF ratio has been proposed as a useful index in the diagnosis and management of PE [22], [23]. Our ELISA assay result (Figure 1) reproduced previous observations [22], [23]. With the range of gestation-week 24 to 40, the control PIGF serum concentrations increased continuously peaked around gestation week 30 and then decreased to the end of the pregnancy. The control sFlt-1 serum concentrations remained relatively stable trending slightly upwards with the gestation weeks. When comparing PE to control subjects, these two analytes' serum concentrations were differentiated with sFlt-1 significantly increased and PIGF significantly decreased throughout the gestation weeks. Our ELISA analysis results provided a sample qualification analysis indicating that our PE and control samples can be used to allow further biomarker discovery and testing analyses.

### PE peptide biomarker identification

**Figure 2:**
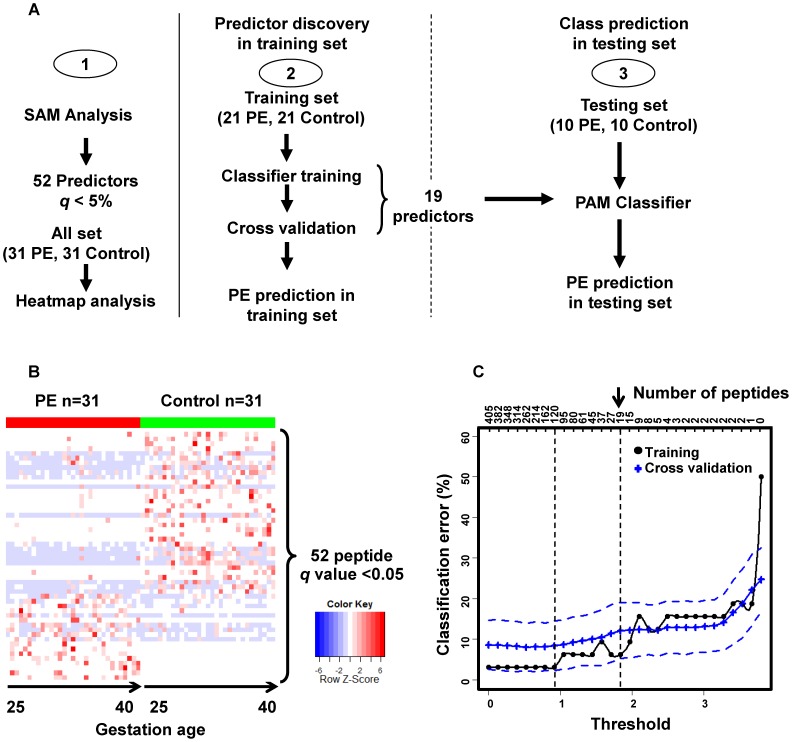
Schematic of the PE serum peptide biomarker discovery and validation. (A) Study outline. (B) Heatmap display of the differential (SAM algorithm, *q*<0.05) serum peptide biomarkers. The rows on the heatmap represent the 52 peptides derived from 14 different proteins with each column of that row representing a different sample from subjects with PE (red) and control (green) subjects. Within PE or control groups, the samples are ordered by gestational age from early to late weeks. (C) Predictor panel discovery by PAM was performed with all the peptide identifications found by LC/MS. In training (black line) and cross-validation (blue line), decreasing the threshold (lower x-axis) resulted in an increase in the number of peptides (inserted upper x-axis) that were used for classification and calculation of the classification error (y-axis). The blue dashed lines represent the variance estimate of predicted error. This led to the discovery of a set of 120 peptides with lowest possible classification error and a minimal practical set of 19 peptides (on the right).

Figure 2A diagrams the PE discriminant peptide biomarker selection, predictive panel construction and validation processes. Initial statistical analysis of the training set by SAM [13] algorithm identified 52 peptides derived from 14 protein precursors with highly significant differences in expression (*q*<5%) between PE and control samples (Table S1). Consistent with the significance findings, heat map plotting (Figure 2B) demonstrated that a differential pattern of the 52 peptides collectively arranged all the samples according to PE and control groups. These results show that the serum abundances of peptide biomarkers are differential between PE and control subjects. In addition, when the heatmap data were sorted according to the gestational age for both PE and control groups, no obvious differential pattern was observed between early and late gestation.

PAM algorithm [14] was used to find a biomarker panel for PE assessment. When constructing the biomarker panel for prediction, there is a trade-off between a small number of selected biomarkers and small prediction errors. As shown in Figure 2C, this minimum error solution (peptide n = 120) might be of interest. Here, to obtain a more manageable set of candidates, a tolerance level of prediction error of 10% and a number of biomarkers (n = 19) were chosen. The selected biomarker panel (Table 3) contains these 19 unique peptides (13 from fibrinogen alpha (FGA), 1 from alpha-1-antitrypsin (A1AT), 1 from apolipoprotein L1 (APO-L1), 1 from inter-alpha-trypsin inhibitor heavy chain H4 (ITIH4), 2 from kininogen-1 (KNG1), and 1 from thymosin beta-4 (TMSB4), totaling 6 protein precursors respectively). All 19 peptide biomarkers have a minimal false discovery rate *q* value<0.05.

**Table 3:** Serum peptide biomarkers identified to separate PE and control subjects.

Proteins	Peptide sequences	MW	Score(d)	*q* value
A1AT	(A)EDPQGDAAQKTDT(S)	1375.06	−1.786	<0.05
APO-L1	(R)VTEPISAESGEQVER(V)	1630.45	1.945	<0.05
FGA*	(R)GSESGIFTNTKESS(S)	1443.27	1.55	<0.05
FGA*	(R)GSESGIFTNTKE(S)	1269.46	4.389	<0.05
FGA*	(G)SESGIFTNTKE(S)	1212.38	2.959	<0.05
FGA**	(K)SYKMADEAGSEADHEGTHST(K)	2123.31	2.95	<0.05
FGA**	(A)DEAGSEADHEGTHST(K)	1543.09	2.135	<0.05
FGA**	(A)DEAGSEADHEGT(H)	1216.75	3.003	<0.05
FGA**	(G)SEADHEGTHST(K)	1169.82	−1.127	<0.05
FGA***	(T)ADSGEGDFLAEGGGV(R)	1379.5	−2.365	<0.05
FGA***	(A)DSGEGDFLAEGGGV(R)	1309.06	−2.41	<0.05
FGA***	(G)DFLAEGGGV(R)	863.416	−1.836	<0.05
FGA****	(Y)NRGDSTFESKSY(K)	1390.73	−3.366	<0.05
FGA****	(Y)NRGDSTFES(K)	1011.8	−2.212	<0.05
FGA****	(G)DSTFESKSY(K)	1063.13	1.647	<0.05
ITIH4	(R)LLGLPGPPDVPDHAAYHPF(R)	2010.71	−2.321	<0.05
KNG-1	(K)LDDDLEHQ(G)	984.17	−2.319	<0.05
KNG-1	(R)IGEIKEETT(V)	1019.3	−1.172	<0.05
TMSB4	(P)SKETIEQEKQAGES(-)	1564.06	−2.745	<0.05

FGA:

* cluster 1;

** cluster 2;

*** cluster 3;

**** cluster 4.

Score and minimal false discovery rate (*q* value) were computed using SAM algorithm.

**Figure 3:**
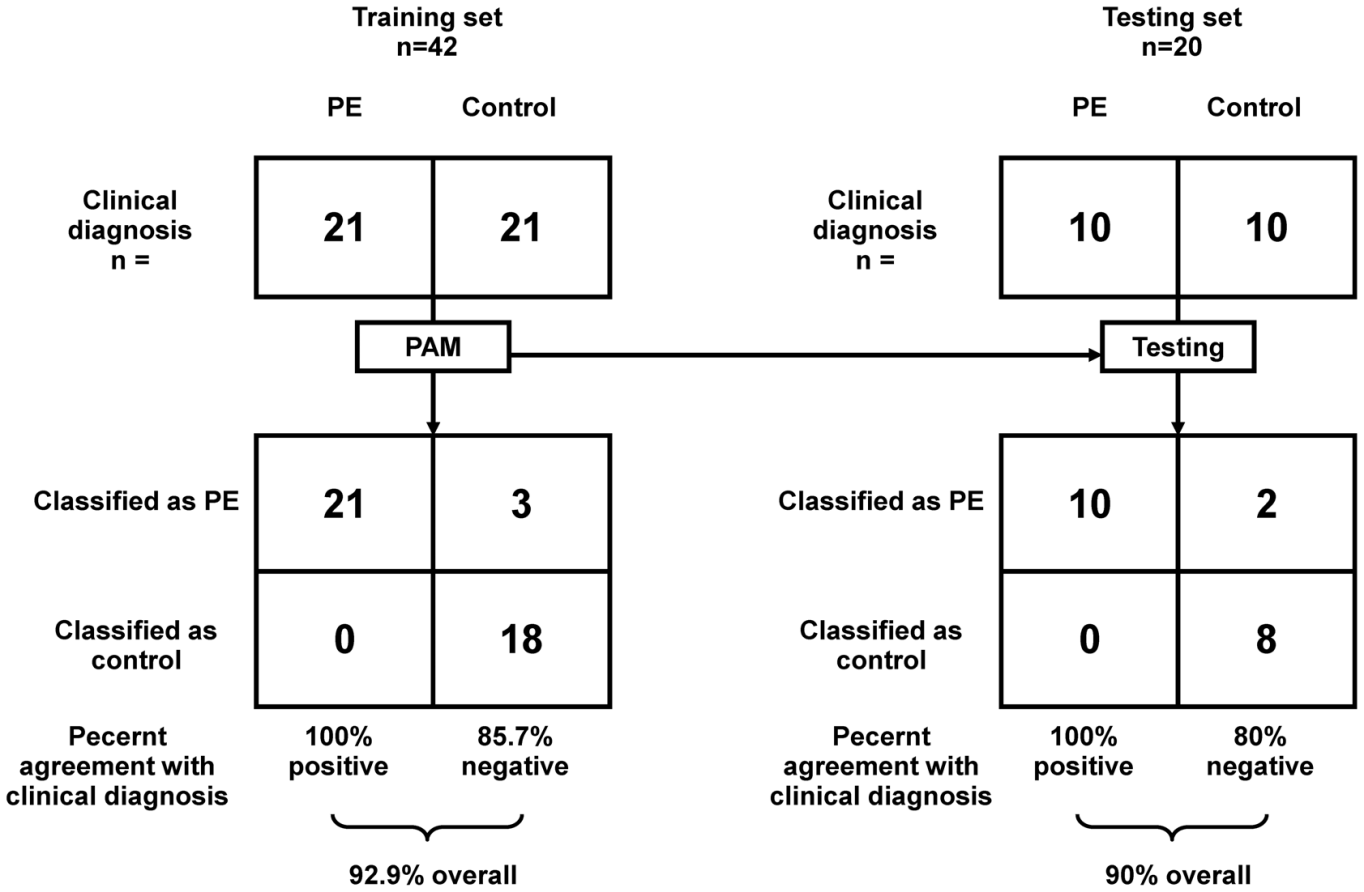
PAM predictive analysis of the 19-peptide biomarker panel differentiating PE from control samples. PAM prediction was performed with training data from PE (training, n = 21; testing, n = 10) and control (training, n = 21; testing, n = 10) samples evaluated with the biomarker panel. Samples are partitioned by the true class (upper) and predicted class (lower). The classification results from training and test sets are shown as 2 by 2 contingency tables, calculating the percentage of classifications that agreed with clinical diagnosis.

**Figure 4:**
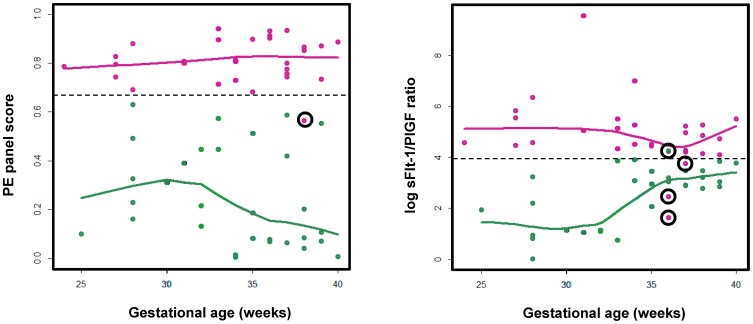
Diagnosis of PE from control with serum biomarkers. Left panel: estimated PE scores were computed from the PE serum peptide panel PAM model as a function of the gestational weeks; right panel: the log sFlt-1/PIGF serum concentration ratio was plotted as a function of the gestational weeks. Red indicates known PE cases; green indicates known healthy pregnancy controls. For either PE or control sample category, a loess curve was fitted to represent the overall trend of biomarker scoring as a function of gestational age.

With the selected biomarker panel and trained PAM prediction model, the PE prediction performance was analyzed as in Figure 3. The left panel of Figure 3 shows the prediction performance on the training set (n = 42), while the right panel of Figure 3 shows the prediction performance on the blind testing set (n = 20). On the training set, all PE samples (n = 21) were predicted correctly, while 3 of the 21 (14.3%) control samples were false positive. Thus, the sensitivity on the training set was 85.7% and the specificity was 100%, resulting in the overall prediction accuracy of 92.9%. Similarly, on the testing set, the overall prediction accuracy is 90%, with sensitivity 80% and specificity 100%. The scatter plot of the PAM predicted scores along with gestational ages is shown as in Figure 4. The predicted score represents the probability of being PE according to the PAM prediction model. Both the prediction accuracy and the scatter plot show that the selected biomarker panel with 19 peptides can be used to effectively predict the occurrence of PE. The early and late gestational age discriminative analyses demonstrated a comparable performance, indicating the potential usefulness of our serum peptide panel in the early diagnosis of PE. The sFlt-1/PIGF ratio's PE assessment utility, previously through the multicenter trial validation [23], was confirmed in this study and used as a benchmark for our newly derived biomarker panels. As shown in Figure 4, the PE diagnostic performance of our peptide panel was comparable to the sFlt-1/PIGF ratio. If we use 0.66, rather than 0.5, as the cutoff of our PE classification panel, as the dotted line in Figure 4, there is only 1 misclassified sample. In contrast with it, the sFlt-1/PIGF ratio results to at least 4 misclassified samples.

### Pathway analysis of PE biomarkers

We analyzed the 14 parental proteins of the 52-peptide markers (found by SAM with *q* value<0.05 that are significantly differentially expressed in PE as a composite), using Ingenuity Pathway Analysis software (IPA version 7.6, Ingenuity Systems, Inc., Redwood City, CA). Our pathway analysis identified the following statistically significant canonical pathways which may play important roles in the pathophysiology of PE: Liver X receptor (LXR)/retinoid X receptor (RXR) activation (*p* value 6.31×10^−19^); atherosclerosis signaling (*p* value 8.31×10^−4^); IL-12 signaling and production in macrophages (*p* value 9.33×10^−9^); clathrin-mediated endocytosis signaling (*p* value 5.89×10^−9^); production of nitric oxide and reactive oxygen species in macrophages (*p* value 6.17×10^−9^); acute phase response signaling (*p* value 2.24×10^−7^); coagulation system (*p* value 3.09×10^−6^); farnesoid X receptor (FXR)/RXR activation (*p* value 7.24×10^−5^); and intrinsic prothrombin activation pathway (*p* value 2.63×10^−4^).

## Discussion

We have employed a serum peptide profiling based approach to identify serum peptide biomarkers that discriminate PE and healthy pregnant controls. 52 significant peptide biomarkers from 14 protein precursors were found and a 19-peptide biomarker panel was constructed which can diagnose PE with great sensitivity and specificity.

The differential 52 serum peptides are derived from proteins known to be involved in the pathophysiology of PE, e.g. A1AT, APO-L1, FGA, ITIH4, KNG1, SERPINA1 in acute inflammatory and defense response; APO-A4, APO-C3, APO-E, and APO-L1 in lipid metabolism; C3, C4A, FGA, and SERPINA1 in the activation of complement and coagulation responses. This might reflect the nature of PE as a multi-factorial disorder with complicated pathophysiological changes. However, little is known about the function of these peptide fragments, including their possible biological activity.

For both systemic and renal diseases, we previously hypothesized [7] that naturally occurring biofluid peptide biomarkers can be the surrogates of pathophysiologies in signaling, proteolytic, and anti-proteolytic pathways. Sequence alignment analyses (Table 3) of these peptides found that FGA peptides line up by forming clusters (n = 4) within either the N- or C-terminal end with ladder-like truncations at the opposite ends, suggesting that there is likely disease-specific proteolytic degradation of the parent protein. The peptide biomarkers can be the derivatives of serological proteins, disease specific shedding from other organs, and/or renal-specific proteins, all of which are generated during the proteolysis that occurs in either circulation during systemic diseases or dysfunctional kidneys, and then trimmed down by exoproteases into ladder-like clusters. The discovery of the serum peptide biomarkers for PE supports the notion that PE pathophysiology or pathogenesis can lead to serum specific protein degradation patterns throughout the progression of the disease from early to late gestation. Moreover, our 19-peptide panel predicted well with comparable sensitivity and specificity at either early or late gestational age weeks, indicating its potential utility throughout the disease course and potentially in early onset of PE. This is in contrast to the established use of the sFlt-1/PIGF ratio [23], which works better in early onset but does not have sufficient statistical power to accurately predict late-onset PE.

Interestingly, we have found an ITIH4 peptide (LLGLPGPPDVPDHAAYHPF) as a PE biomarker. This peptide shares an almost identical sequence as a previously published spontaneous preterm birth (SPB) serum peptide biomarker (QLGLPGPPDVPDHAAYHPF) [24] but there is a preceding amino acid sequence change from L to Q [24]. Close examination of a database of common gene variations (http://snp.ims.u-tokyo.ac.jp/cgi-bin/SnpInfo.cgi?SNP_ID=IMS-JST073530) revealed that this change is due to the single nucleotide polymorphism (SNP) in ITIH4 where a single coding nucleotide differs from A of amino acid codon cAa to T of cTa, resulting in an amino acid change from Q to L. The exact biological function of ITIH4 and its degraded serum peptide is unknown. Given that the same ITIH4 peptide is a biomarker of both PE and SPB, it is very likely that this is not a disease-process-related biomarker as PE and SPB have very different pathophysiologies.

We also recognize several limitations to our study. Proteomic profiling data were acquired from a commercial vendor with little specific information on the clinical characteristics including blood pressure at the time of delivery, baseline blood pressure, birth weight, level of proteinuria which are data that one would normally see in a study on preeclampsia. Current analysis can only be of confirmative diagnostic rather than predictive values. Samples at asymptomatic stages of pregnancy (i.e. at earlier time points) should have been examined to study the predictive value of the panel. Samples from women with other hypertensive disorders of pregnancy would be required to see if the panel differentiates between these and PE. Both of these aspects would be clinically relevant. The former in order to target intensive monitoring and preventative strategies to those at risk, the latter in order to target therapy (i.e. delivery of the baby) to those with PE whereas women with other hypertensive disorders could potentially continue with their pregnancy. In addition, there is heavy bias towards African American, Asian and Hispanic ethnicities. Robust prospective analysis of this 19-peptide panel in sufficiently powered independent samples would still be mandatory to validate this panel's clinical usefulness in PE diagnosis.

We proposed that serum peptidome biomarker analysis might be useful in diagnosing PE, however, the challenges in developing cleavage site-specific antibodies and a resultant ELISA for these peptide biomarkers make it difficult for translation into a point-of-care antibody-based assay. Technologic advances in multiple reaction monitoring (MRM) [25], [26] coupled with stable isotope dilution (SID) mass spectrometry (MS) have empowered a “universal” approach to perform quantitative assays for peptides with minimum restrictions, and the ease of assembling multiplex peptide detections in a single measurement. Using common materials and standardized protocols, the reproducibility and transferability of MRM assays between laboratories and across instrument platforms have been demonstrated [25]. Therefore, in a similar fashion as the current common practice of applying MRM based newborn screening of metabolic diseases, a greater acceptance by the clinical community of SID-MRM-MS technology as a generally applicable approach for biofluid protein and peptide quantification is expected. We believe a future prospective trial of our serum peptide PE biomarker panel, using SID-MRM-MS, will lead to a quick and reliable multiplexed test which can be run routinely in the hospital setting for PE care.

## Supporting Information

Table S1 **Serum peptides identified by SAM algorithm (** ***q*** **value<0.05), which are significantly differentiated between PE and control subjects.**
(PDF)
