# Supplementary material for: Peptidomic Identification of Serum Peptides Diagnosing Preeclampsia
Source: PLoS One. 2013 Jun 19;8(6):e65571. doi: 10.1371/journal.pone.0065571 (PMC3686758; doi:10.1371/journal.pone.0065571)
Supplement: Table S1 — Serum peptides identified by SAM algorithm ( q value<0.05), which are significantly differentiated between PE and control subjects. (PDF) [file pone.0065571.s001.pdf]

| Supplementary Table 1. Serum peptides identified by SAM algorithm (*q* value<0.05) which are significantly differentiated between PE and control subjects. | | | | |
|---|---|---|---|---|
| Heatmap index | Protein | Peptide sequence | Score(d) | *q* value(%) |
| 1 | FGA | (R)GSESGIFTNTKE(S) | 6.141762 | 0 |
| 2 | FGA | (G)SEADHEGTHST(K) | 5.186152 | 0 |
| 3 | KNG1 | (K)LDDDLEHQ(G) | 3.857129 | 0 |
| 4 | TMSB4 | (P)SKETIEQEKQAGES(-) | 3.688479 | 0 |
| 5 | FGA | (G)SESGIFTNTKE(S) | 3.622314 | 0 |
| 6 | C3 | (R)SEETKENEGFTV(T) | 3.536669 | 0 |
| 7 | TMSB4 | (S)KETIEQEKQAGES(-) | 3.478967 | 0 |
| 8 | APO-A4 | (G)NTEGLQ(K) | 3.369214 | 0 |
| 9 | FGA | (A)DEAGSEADHEGTH(S) | 3.364307 | 0 |
| 10 | FGA | (E)GDFLAEGGGV(R) | 3.255781 | 0 |
| 11 | FGA | (A)DEAGSEADHEGT(H) | 3.157053 | 0 |
| 12 | FGA | (R)GSESGIFTNTKESS(S) | 3.10426 | 0 |
| 13 | FGA | (A)DEAGSEADHEGTHST(K) | 2.973072 | 0 |
| 14 | APO-E | (A)TVGSLAG(Q) | 2.874127 | 0 |
| 15 | TMSB4 | (K)ETIEQEKQAGES(-) | 2.643713 | 0 |
| 16 | APO-A4 | (L)GGHLDQQVEEF(R) | 2.6235 | 0 |
| 17 | APO-C3 | (S)SVQESQVAQQA(R) | 2.567146 | 0 |
| 18 | ITIH4 | (R)LLGLPGPPDVPDHAAYHPF(R) | 2.554118 | 0 |
| 19 | APO-L1 | (R)VTEPISAESGEQVER(V) | 2.520311 | 0 |
| 20 | C3 | (R)SEETKENEGF(T) | 2.504033 | 0 |
| 21 | FGA | (G)SESGIFTNTKESS(S) | 2.409848 | 1.893749 |
| 22 | APO-E | (L)DEVKEQVAEV(R) | 2.392038 | 1.893749 |
| 23 | ZYX | (R)GPPASSPAPAPK(F) | 2.34915 | 1.893749 |
| 24 | KNG1 | (R)IGEIKEETT(V) | 2.341402 | 1.893749 |
| 25 | C3 | (R)SEETKENEGFTVTAEGK(G) | 2.305142 | 1.893749 |
| 26 | APO-A1 | (R)LEALKENGGA(R) | 2.304021 | 1.893749 |
| 27 | APO-C3 | (K)TAKDALSSVQES(Q) | 2.296693 | 1.893749 |
| 28 | C3 | (I)HWESASL(L) | 2.235505 | 3.2824982 |
| 29 | APO-A4 | (I)DQNVEELKG(R) | 2.232068 | 3.2824982 |
| 30 | KNG1 | (K)LDDDLEHQGGHVLDHGH(K) | 2.210918 | 3.2824982 |
| 31 | FGA | (A)DEAGSEADHEGTHSTKR(G) | 2.179585 | 3.2824982 |
| 32 | HRNR | (Y)GSGSGWSSSRGPY(E) | 2.132342 | 3.2824982 |
| 33 | C4A | (R)TLEIPGN(S) | 2.119886 | 4.2609351 |
| 34 | APO-E | (A)VGTSAAPVPSDNH(-) | 2.083623 | 4.2609351 |
| 35 | FGA | (Y)NRGDSTFES(K) | -3.7179 | 0 |
| 36 | FGA | (D)FLAEGGGV(R) | -3.39524 | 0 |

| | | | | |
|---|---|---|---|---|
| 37 | FGA | (T)SYNRGDSTFES(K) | -3.2551 | 0 |
| 38 | FGA | (Y)NRGDSTFESKS(Y) | -3.2183 | 0 |
| 39 | FGA | (D)STFESKSY(K) | -2.91552 | 0 |
| 40 | SERPINA1 | (A)EDPQGDAAQKTDT(S) | -2.79146 | 0 |
| 41 | FGA | (G)DFLAEGG(G) | -2.74576 | 0 |
| 42 | FGA | (G)EGDFLAEGGGV(R) | -2.73961 | 0 |
| 43 | FGA | (G)EGDFLAEGGG(V) | -2.73489 | 0 |
| 44 | FGA | (K)MADEAGSEADHEGTHST(K) | -2.68688 | 2.3824584 |
| 45 | FGA | (G)DFLAEGGGV(R) | -2.57087 | 2.3824584 |
| 46 | FGA | (G)DSTFESKSY(K) | -2.52742 | 2.3824584 |
| 47 | FGA | (Q)FTSSTSYNRGDSTFES(K) | -2.42019 | 3.2824982 |
| 48 | FGA | (A)DSGEGDFLAEGGGV(R) | -2.41329 | 3.2824982 |
| 49 | FGA | (K)SYKMADEAGSEADHEGTHST(K) | -2.28074 | 4.2609351 |
| 50 | FGA | (G)DFLAEGGGVR(G) | -2.27309 | 4.2609351 |
| 51 | FGA | (S)YKMADEAGSEADHEGTHST(K) | -2.26121 | 4.2609351 |
| 52 | FGA | (G)DFLAEGGG(V) | -2.23299 | 4.2609351 |
